# Umbelliferone Prevents Lipopolysaccharide-Induced Bone Loss and Suppresses RANKL-Induced Osteoclastogenesis by Attenuating Akt-c-Fos-NFATc1 Signaling

**DOI:** 10.7150/ijbs.28609

**Published:** 2019-09-07

**Authors:** Sung Chul Kwak, Jong Min Baek, Chang Hoon Lee, Kwon-Ha Yoon, Myeung Su Lee, Ju-Young Kim

**Affiliations:** 1 Department of Anatomy, School of Medicine, Wonkwang University, Iksan, Jeonbuk 570-749, Republic of Korea; 2 HUONS Research Center, Hanyang University in ERICA campus, Ansan 15588, Republic of Korea; 3 Division of Rheumatology, Department of Internal Medicine, Wonkwang University, Iksan, Jeonbuk 570-749, Republic of Korea; 4 Department of Radiology, School of Medicine, Wonkwang University, Iksan, Jeonbuk 570-749, Republic of Korea; 5 Medical Convergence Research Center, Wonkwang University Hospital, 460 Iksandae-ro, Iksan, Jeonbuk 570-749, Republic of Korea

**Keywords:** umbelliferone, inflammatory bone diseases, osteoclast, Akt-c-Fos-NFATc1 signaling, bone resorption

## Abstract

Excessive bone resorption plays a central role in the development of inflammatory bone diseases, including osteoporosis and rheumatoid arthritis. Thus, identification of agents that can effectively suppress excessive osteoclast formation and function is crucial for the prevention and treatment of inflammatory bone loss. Umbelliferone (Umb), a derivative of coumarin, is a natural bioactive compound with anti-inflammatory and antioxidant properties. However, the effect of Umb on metabolic bone diseases is unknown. In this study, we found that Umb exhibited a strong inhibitory effect on lipopolysaccharide (LPS)-induced inflammatory bone loss *in vivo*. Histological analysis confirmed that Umb prevented trabecular bone matrix degradation and osteoclast formation in bone tissue. In addition, Umb suppressed RANKL-induced osteoclast differentiation and abrogated bone resorption. We found that the anti-osteoclastic and anti-resorptive activities of Umb are mediated *via* suppression of the RANKL-induced Akt-c-Fos-NFATc1 signaling pathway and the attenuation of osteoclast-specific genes, such as *TRAP*,* OSCAR*,* ATP6v0d2*, and *CtsK*. In particular, Umb downregulated the stability of c-Fos and NFATc1 proteins, but did not suppress the expression of their mRNAs. These results indicate that Umb may be a potential therapeutic agent for inflammatory bone diseases associated with abnormal osteoclast formation and function.

## Introduction

Bone destruction, known as osteolysis, is characterized by excessive osteoclastic bone resorption and occurs in several inflammatory conditions. Among the pathological factors contributing to the frequent incidence of osteoporosis, increased secretion of pro-inflammatory cytokines such as interleukin (IL)-1, IL-6, IL-17, and tumor necrosis factor-alpha (TNF-α) suggests an intimate relationship between inflammation and osteoporosis 1-3. Hence, a number of reports have focused on various inflammatory diseases implicated in osteoporosis. In comparison with the general population, patients with inflammatory bowel disease have increased rates of osteoporosis and osteopenia due to the following conditions: use of corticosteroids; malabsorption of vitamin D, vitamin K, and calcium; and hypogonadism 4. Furthermore, rheumatoid arthritis, a typical inflammatory joint disease, induces several features of bone loss, including focal bone erosion, subchondral osteolysis, periarticular osteopenia, and axial and appendicular osteopenia 5. To prevent these inflammatory bone diseases, a number of experimental studies in mice aimed to discover unidentified osteoporosis-preventing agents by studying the effects of various compounds on bone loss induced by lipopolysaccharide (LPS), a component of the outer membranes of gram-negative bacteria 6. As a prominent inducer of inflammatory bone erosion, LPS promotes both the survival of osteoclasts responsible for bone resorption through toll-like receptor 4 (TLR4) and RANKL-mediated osteoclast differentiation, creating a pathologic microenvironment that easily contributes to bacteria-derived osteolysis 6-9.

The differentiation of monocyte/macrophage lineage cells into osteoclasts (multinucleated bone-resorbing cells) occurs in response to two essential cytokines: macrophage colony-stimulating factor (M-CSF) and receptor activator of nuclear factor kappa-B ligand (RANKL) 10. RANKL is a member of the tumor necrosis family (TNF) and activates intracellular signals of osteoclastogenesis, including mitogen-activated protein kinase (MAPK) and nuclear factor kappa-light-chain-enhancer of activated B cells (NF-κB) 11. These two distinct signaling pathways activate nuclear factor of activated T-cells cytoplasmic 1 (NFATc1), which is a key transcription regulator of osteoclast differentiation, inducing the release of a number of osteoclast-specific genes, such as tartrate-resistant acid phosphatase (*TRAP*), osteoclast-associated receptor (*OSCAR*), and cathepsin K (*CtsK*) 12, 13.

Numerous medicinal plants have been cultivated to develop novel therapies for metabolic bone disorders. Umbelliferone (Umb) is a natural compound belonging to the coumarin family, extracted not only from Umbelliferae plants, including carrot, angelica, and coriander but also from members of other plant families, such as *Gmelina arborea* 14, 15. As a coumarin derivative, Umb is regarded as a promising substance in the management of various pathological conditions, especially those involving oxidative stress 16-18. Umb also has antinociceptive and anti-inflammatory properties 18-20. It is well established that anti-inflammatory and anti-oxidant properties are closely associated with the dysfunction of osteoclasts attributed to defects in osteoclast survival and differentiation, suggesting that Umb may be effective in the treatment of diseases related to bone metabolism. Thus, in the current study, Umb was orally administered to mice with LPS-mediated systemic inflammatory bone loss to determine whether Umb inhibited abnormal bone resorption *in vivo*. To support the *in vivo* study, we also investigated the effects of Umb on osteoclast formation and function during RANKL-mediated osteoclastogenesis and its underlying intracellular signaling pathways *in vitro*.

## Materials and Methods

### Mice

Five-week-old male ICR mice were purchased from Samtako Bio Korea (Osan, South Korea). The mice were kept in a temperature- (22°C to 24°C) and humidity (55% to 60%)-controlled environment with a 12-hour light/dark cycle. All experiments were conducted according to the guidelines of the Institutional Animal Care and Use Committee (IACUC) of Wonkwang University (approval number: WKU17-01).

### Reagents and antibodies

Umb was purchased from Sigma-Aldrich (St. Louis, MO, USA). The Umb was prepared as a 200 mM stock in dimethyl sulfoxide (DMSO) and stored at -20°C. The Umb was added to cell culture medium such that the DMSO comprised < 0.1% of the volume of the culture medium. For *in vivo* study, Umb was dissolved in 0.1% carboxymethyl cellulose (CMC) and administered orally. 1,25-dihydroxyvitamin D_3_ (VitD_3_), prostaglandin E_2_ (PGE_2_), ascorbic acid (AA), beta-glycerol phosphate (β-GP), LPS, and a monoclonal β-actin antibody were obtained from Sigma-Aldrich (St. Louis, MO, USA). Soluble recombinant human M-CSF and human RANKL were obtained from PeproTech EC, Ltd. (London, UK). Cycloheximide (CHX) and MG132 were obtained from Calbiochem (San Diego, CA, USA). Anti-p38, anti-phospho-p38, anti-JNK, anti-phospho-JNK, anti-Akt, anti-phospho-Akt, anti-IκB, anti-phospho-IκB, anti-Bruton's tyrosine kinase (Btk), and anti-phospho-Btk antibodies were purchased from Cell Signaling Technology, Inc. (Beverly, MA, USA). Anti-c-Fos, anti-NFATc1, anti-phospholipase C gamma 2 (PLCγ2), and anti-phospho-PLCγ2 antibodies were purchased from Santa Cruz Biotechnology (Santa Cruz, CA, USA). Fetal bovine serum (FBS), α-minimum essential medium (α-MEM), and penicillin/streptomycin were purchased from Gibco BRL (Grand Island, NY, USA). All other chemicals were of analytical grade or complied with the standards required for cell culture.

### *In vitro* osteoclastogenesis assay

Bone marrow cells (BMCs) were obtained from five-week-old male ICR mice by flushing their femurs and tibias with α-MEM supplemented with 10% FBS, penicillin (100 U/mL), and streptomycin (100 μg/mL). To obtain bone marrow macrophages (BMMs), BMCs were seeded on culture dishes in α-MEM supplemented with 10% FBS and macrophage colony stimulating factor (M-CSF; 10 ng/mL) and cultured for one day. Nonadherent cells were transferred to 10 cm petri dishes and further cultured in the presence of M-CSF (30 ng/mL) for three days. After the nonadherent cells were removed, the adherent cells were used as BMMs, which are osteoclast precursors. To generate osteoclasts from these BMMs, the cells were seeded in a 48-well plate (3.5 × 10^4^ cells/well) in complete medium containing M-CSF (30 ng/mL) and RANKL (100 ng/mL) and cultured for four days with or without Umb. The cells were fixed in 3.7% formalin for 10 minutes, permeabilized with 0.1% Triton X-100, and then stained with TRAP staining solution (0.1 mg/mL naphthol AS-MX phosphate and 0.3 mg/mL Fast Red Violet LB salt). TRAP-positive multinucleated cells (MNCs) with more than three nuclei were counted as osteoclasts.

### Cytotoxicity assay

An XTT {sodium 30-[1-(phenyl-aminocarbonyl)-3,4-tetrazolum]-bis(4-methoxy-6-nitro) benzenesulfonic acid hydrate and N-methyl dibenzopyrazine methyl sulfate} assay was performed to examine the effects of Umb on the viability of BMMs. BMMs (1 × 10^4^ cells/well) were seeded in 96-well plates with various concentrations of Umb and incubated for 3 days in the presence of M-CSF (30 ng/mL). Then, XTT solution (50 μL) was added to each well and incubated for 4 hours. The plate was read at 450 nm with an ELISA reader (Molecular Devices, Sunnyvale, CA, USA).

### Pit formation assay

BMCs (1 × 10^7^ cells) and primary osteoblasts (1 × 10^6^ cells) were seeded on collagen gel-coated culture dishes and cultured for 7 days in the presence of 10 nM VitD_3_ and 1 μM PGE_2_. The co-cultured cells were detached by 0.1% collagenase treatment at 37°C for 10 minutes and were then re-plated on hydroxyapatite-coated plates (Corning, Corning, NY, USA). The cells were incubated on the plates with or without Umb. After 24 hours, the cells were removed and the total area of all resorption pits was photographed and analyzed using Image-Pro Plus software version 4.0 (Media Cybernetics, Rockville, MD, USA).

### Osteoblastic cell culture and assays

To culture osteoblasts, the calvariae of neonatal mice were digested with 0.1% collagenase and 0.2% dispase five times; the cells isolated in the last three digestions were combined and cultured in α-MEM containing 10% FBS, 100 U/mL penicillin, and 100 μg/mL streptomycin. To measure alkaline phosphatase (ALP) activity, primary osteoblasts were inoculated at a density of 2 × 10^4^ cells/well and cultured in the absence or presence of 50 μg/mL AA and 10 mM β-GP. On day six of differentiation, the cells were sonicated in 50 mM Tris-HCl buffer (pH 7.4) containing 1% Triton X-100, 150 mM NaCl, and 1 mM EDTA. Then, 100 μL of substrate (*p*-nitrophenylphosphate) (Sigma-Aldrich) was added to the cells and the plate was incubated for 30 minutes at 37°C. The amount of *p*-nitrophenol released was determined by measuring the absorbance at 405 nm using a microplate reader. For ALP staining, cells were fixed in 70% ethanol and stained for 10 minutes with a solution containing 0.01% naphthol AS-MX phosphate, 1% N,N-dimethylformamide, and 0.06% Fast Blue BB (Sigma-Aldrich). Alizarin Red S (ARS) staining was performed on day 21 of differentiation; cultured cells were fixed in 3.7% formalin and stained for 10 minutes with 2% ARS (pH 4.2) (Sigma-Aldrich). The bound ARS was dissolved in a 10% cetylpyridinium chloride (CPC) monohydrate solution (pH 7.0). Absorbance was measured at 545 nm using a microplate reader.

### Real-time quantitative reverse transcription PCR

Total RNA was isolated with TRIzol reagent (Life Technologies, Carlsbad, CA, USA) according to the manufacturer's instructions. To obtain cDNA, equal amounts of total RNA were reverse-transcribed into cDNA using RevertAid Reverse Transcriptase (ThermoFisher Scientific, Waltham, MA, USA). Real-time RT-qPCR was performed in a 20 µL reaction mixture containing 10 µL of SYBR Green Premix (Bioneer Co., Daejeon, South Korea), 10 pmol of forward primer, 10 pmol of reverse primer, and 100 ng of cDNA using an Exicycler™ 96 Real-Time Quantitative Thermal Block (Bioneer Co.). The primers used to detect the genes of interest were as follows*: c-Fos,* forward 5'-GGTGAAGACCGTGTCAGGAG-3' and reverse 5'-TATTCCGTTCCCTTCGGATT-3'; *NFATc1*, forward 5'-GAGTACACCTTCCAGCACCTT-3' and reverse 5'-TATGATGTCGGGGAAAGAGA-3'; *OSCAR*, forward 5'-GGAATGGTCCTCATCTGCTT-3' and reverse 5'-GGAATGGTCCTCATCTGCTT-3'; *TRAP*, forward 5'-TCATGGGTGGTGCTGCT-3' and reverse 5'-GCCCACAGCCACAAATCT-3'; *ATP6v0d2*, forward 5'-GACCCTGTGGCACTTTTTGT-3' and reverse 5'-GTGTTTGAGCTTGGGGAGAA-3'; *CtsK*, forward 5'-CCAGTGGGAGCTATGGAAGA-3' and reverse 5'-CTCCAGGTTATGGGCAGAGA-3';* Runx2*, forward 5'-TGCCTTCAGCACCCTATACC-3' and reverse 5'-AGGTTGGAGGCACACATAGG-3'; *ALP*, forward 5'-GCTGATCATTCCCACGTTTT-3' and reverse 5'-ACCATATAGGATGGCCGTGA-3'; bone sialoprotein (*BSP*), forward 5'-AGGGAACTGACCAGTGTTG-3' and reverse 5'-ACTCAACGGTGCTGCTTTTT-3', collagen type I alpha 1 (*COL1A1*) forward 5'-TGTGTTCCCTACTCAGCCGTCT-3' and reverse 5'-CATCGGTCATGCTCTCTCCAA-3'; and glyceraldehyde-3-phosphate dehydrogenase (*GAPDH*), forward 5'-TCAAGAAGGTGGTGAAGCAG-3' and reverse 5'-AGTGGGAGTTGCTGTTGAAGT-3'. The mouse *GAPDH* gene was used as an internal control. The amplification parameters consisted of an initial denaturation step at 95°C for 5 minutes, followed by 40 cycles of denaturation at 95°C for 15 seconds and annealing/extension at 60°C for 30 seconds. The specificity of the SYBR green assays was confirmed by melting-point analysis. Expression data were calculated from the cycle threshold (Ct) value using the Ct method.

### Retrovirus preparation and infection

Packaging of the retroviral vectors pMX-IRES-EGFP and pMX-constitutively active (CA)-Akt-IRES-EGFP was performed by transient transfection of these pMX vectors into Plat-E retroviral packaging cells using X-tremeGENE 9 (Roche, Nutley, NJ, USA) according to the manufacturer's protocol. After incubation in fresh medium for 2 days, the culture supernatants of the retrovirus-producing cells were collected. For retroviral infection, nonadherent BMCs were cultured in M-CSF (30 ng/mL) for 2 days. The BMMs were incubated with viral supernatant pMX-IRES-EGFP and pMX-Akt-IRES-EGFP virus-producing Plat-E cells together with polybrene (10 μg/mL) and M-CSF (30 ng/mL) for 6 hours. The infection efficiency of the retroviruses was determined by green fluorescent protein expression; these assessments yielded efficiencies greater than 80%. After infection, the BMMs were induced to differentiate on medium with or without Umb in the presence of M-CSF (30 ng/mL) and RANKL (100 ng/mL) for three days. Osteoclast formation was detected by TRAP staining.

### Stability of c-Fos and NFATc1

The BMMs were incubated for 6 hours with c-Fos or NFATc1 retroviral soup produced by Plat-E cells together with polybrene (10 μg/mL) and M-CSF (10 ng/mL). Infected BMMs were pretreated with or without Umb in the presence of M-CSF for 24 hours, then stimulated with RANKL. After 20 hours, 2 μg/mL CHX and/or 5 mM MG132 were added to the cultures 4 hours before harvesting.

### Western blot analysis

Whole-cell lysates were prepared using lysis buffer containing 50 mM Tris-HCl, 150 mM NaCl, 5 mM EDTA, 1% Triton X-100, 1 mM sodium fluoride, 1 mM sodium vanadate, 1% deoxycholate, and protease inhibitors. The suspension was centrifuged at 14,000 g for 20 minutes and the supernatant was collected. The protein content was measured using a DC Protein Assay Kit (Bio‐Rad Laboratories, Inc., Hercules, CA, USA). Equal amounts of protein (20 µg) were electrophoresed on 8% to 10% SDS-PAGE gels and transferred by electroblotting onto polyvinylidene difluoride membranes (MilliporeSigma, Bedford, MA, USA). The membranes were blocked with 5% nonfat milk in Tris-buffered saline and 0.1% Tween-20 (TBST) for one hour, before blotting with the primary antibodies for 2 hours at room temperature. The membranes were washed in TBST and incubated for 1 hour with horseradish peroxidase-conjugated sheep anti-mouse or donkey anti-rabbit immunoglobulin antibodies. Specific signals were detected using Western Chemiluminescent HRP substrate (MilliporeSigma).

### LPS-induced inflammatory bone loss model and analysis

To study the effect of Umb on LPS-induced inflammatory bone loss *in vivo*, ICR mice were divided into four experimental groups composed of five mice each: the 1% CMC-treated (control), only LPS-treated, and LPS with two concentrations of Umb-treated groups. Umb (60 and 120 mg/kg) or 1% CMC was administered orally as control one day before LPS injection (5 mg/kg). Umb or 1% CMC was administered orally every other day for eight days. LPS was injected intraperitoneally on days one and four. Body weight and food intake were monitored daily. The mice were euthanized after eight days and their left and right femurs were analyzed by high-resolution microcomputed tomography (micro-CT). The metaphyseal regions of the femurs were scanned using a high-resolution micro-CT (NFR-Polaris S160; Nanofocus Ray, Iksan, South Korea) with a source voltage of 50 kVp, current of 70 µA, and 6 µm isotropic resolution. Femur scans were performed proximal to the growth plate on 2-mm-thick cross-sections, with 720 sections per scan. Bone histomorphometric analysis was carried out on the micro-CT data using INFINITT‐Xelis software (INFINITT Healthcare, Seoul, South Korea). The structural parameters were trabecular bone volume/total volume (BV/TV, %), trabecular thickness (Tb.Th, μm), trabecular separation (Tb.Sp, μm), and trabecular number (Tb.N, 1/mm). After micro-CT analysis, the femurs were fixed in 4% paraformaldehyde (Sigma-Aldrich) for one day, decalcified for three weeks in 12% EDTA, and embedded in paraffin. Sections (5 µm thick) were prepared using a Leica microtome RM 2145 (Leica Microsystems, Bannockburn, IL, USA) and stained with hematoxylin and eosin (H&E). Other sections were stained with TRAP to visualize osteoclasts. Parameters for bone resorption included eroded surface per bone surface (ES/BS, %), number of osteoclasts per field of tissue were quantified by using the Image Pro-Plus program, version 4.0 (Media Cybernetics, Silver Spring, MD). Serum CTX-I levels, a specific marker of bone resorption, were determined using a mouse-specific ELISA assay according to the manufacturer's protocol (Nordic Bioscience Diagnostics, Herlev, Denmark).

### Statistical analysis

Experiments were conducted at least three times and the data are expressed as mean ± standard deviation (SD). All statistical analysis was performed using the Statistical Package for the Social Sciences Software (SPSS, Korean version 14.0; Chicago, IL, USA). The statistical differences were analyzed using one-way ANOVA followed by the Tukey post-hoc test. *P* < 0.05 was considered statistically significant.

## Results

### Umb prevents LPS-induced inflammatory bone loss* in vivo*

**Fig 1:**
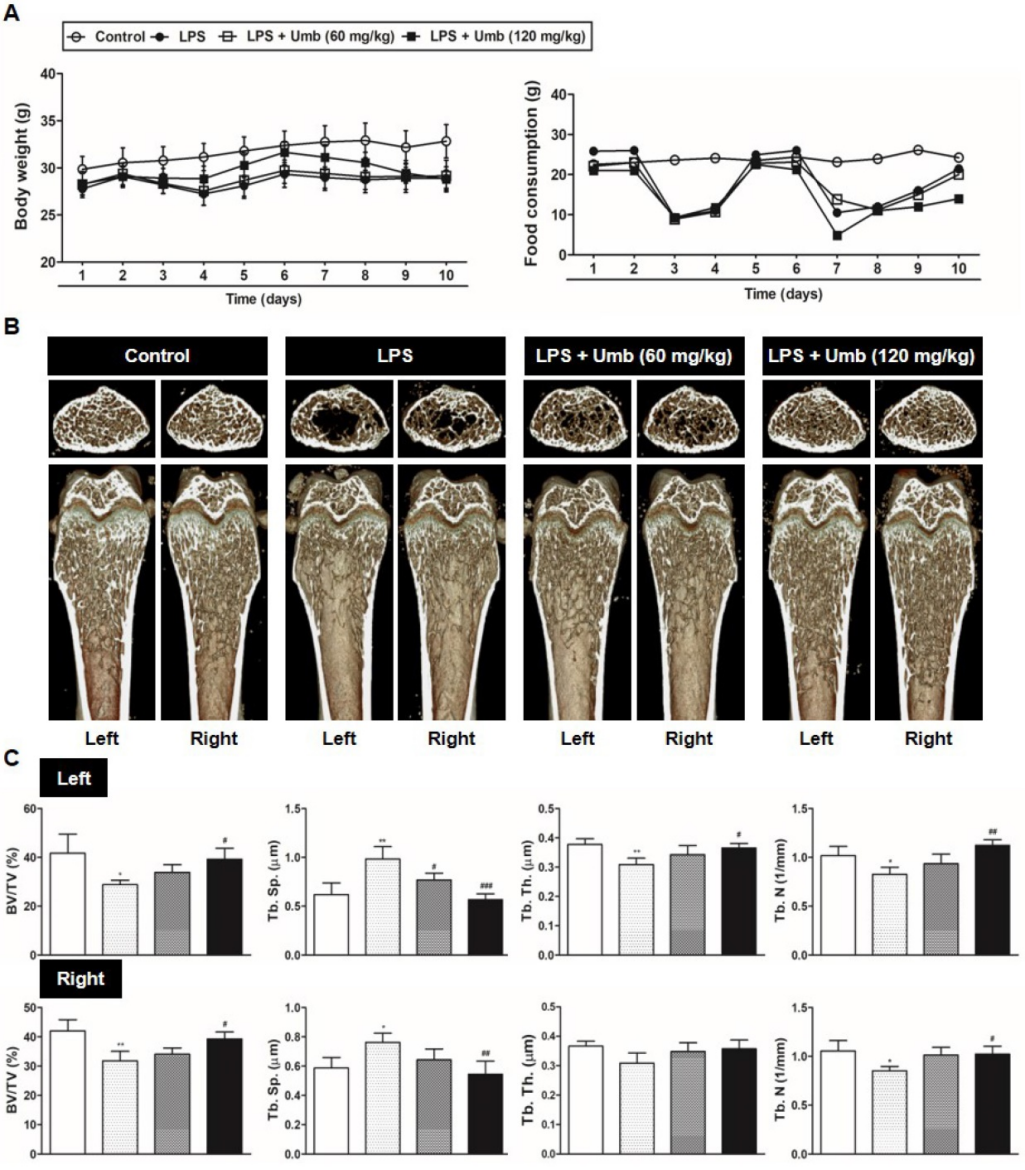
Umb prevents LPS-induced inflammatory bone loss *in vivo.* (A) Umb (60, 120 mg/kg) or 1% CMC was administered orally as control 1 day before LPS injection (5 mg/kg). Umb or 1% CMC was administered orally every other day for 8 days. LPS was injected intraperitoneally on days 1 and 4. Bodyweight and food consumption were measured during the experimental period. (B) Mice were euthanized 8 days after the first LPS injection and radiographs of longitudinal and transverse sections of the proximal femur were obtained with a micro-CT apparatus. (C) The BV/TV, Tb.Sp, Tb.Th, and Tb.N of left and right femurs were determined using the micro-CT data as analyzed with INFINITT-Xelis software.

**Fig 2:**
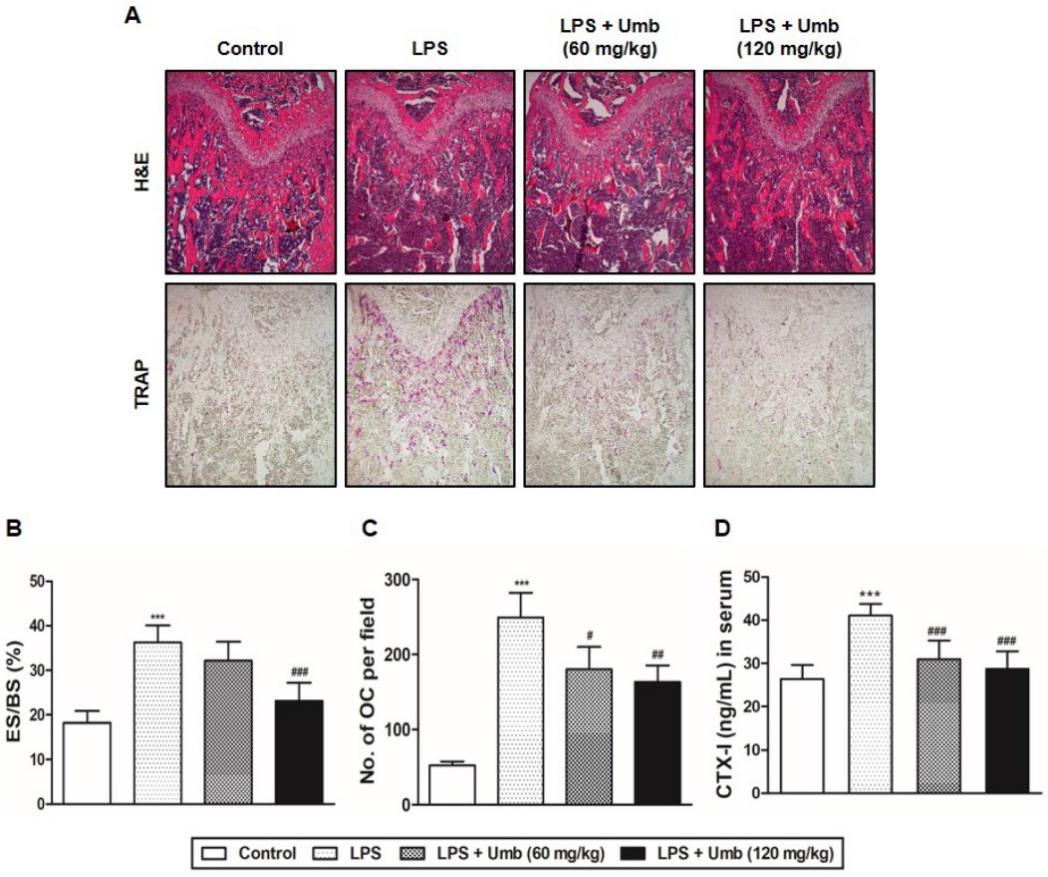
Umb inhibits inflammatory osteolysis of bone matrix and the number of TRAP-positive cells in LPS-induced inflammatory bone loss based on histological analysis. (A) Mice were sacrificed 8 days after the first LPS injection. Dissected femora were fixed, decalcified, embedded, and sectioned. Sections were stained with H&E (*upper*) and with TRAP (*lower*). (B) Eroded surface per bone surface (ES/BS, %) and (C) the number of osteoclasts per field of tissue was analyzed using the histomorphometric results. (D) Measurement of CTX-I in serum of controls and mice treated with LPS, or LPS plus Umb (60 or 120 mg/kg) by ELISA. ^***^P < 0.001 versus control group; *^#^P* < 0.05, *^##^P* < 0.01, *^###^P* < 0.001versus LPS group.

To explore the effect of Umb on pathological osteolysis *in vivo*, we used an LPS-induced inflammatory bone loss model. We investigated the toxicity of Umb in the mice by measuring food intake and bodyweight. The Umb-treated group exhibited neither any symptoms of toxicity nor other significant differences when compared to the control group (Fig. 1A). No fatalities were recorded after LPS and Umb administration and the animals maintained normal activity throughout the duration of the experiment. Micro-CT scanning and 3D reconstruction of the left- and right-femurs revealed that LPS injection (LPS group) resulted in bone loss in the trabecular bone of femurs relative to 1% CMC injection (control group). However, treatment with Umb (60 mg/kg-dose group and 120 mg/kg-dose group) strongly suppressed LPS-induced inflammatory osteolysis (Fig. 1B). Quantitative analysis of bone parameters further demonstrated that LPS-induced decreases in BV/TV, Tb.Th, and Tb.N were largely reversed by treatment with Umb 120 mg/kg (Fig. 1C). Additionally, Tb.Sp was significantly higher in the LPS group; it was decreased by Umb (Fig. 1C). Histological assessment confirmed the prevention effect of Umb on LPS-induced osteolysis. Images of the H&E-stained histological sections showed bone matrix with increased osteolysis in the LPS**-**induced group compared with that in the control group, whereas bone matrix in the Umb-treated LPS**-**induced group was restored (Fig. 2A, *upper* and Fig. 2B). Consistent with the pronounced reduction in inflammatory osteolysis, the number of TRAP-positive cells observed in the LPS-induced group was markedly reduced by Umb treatment (Fig. 2A, *lower* and Fig. 2C). Also, the serum C-teminal telopeptide of type I collagen (CTX-I) concentration, a bone resorption marker, were substantially higher in the LPS treated group. The serum level of CTX-I increased by LPS is effectively declined by treating Umb (60 mg/kg-dose group and 120 mg/kg-dose group) (Fig. 2D). These results demonstrate that Umb protects against LPS-induced inflammatory bone loss without causing toxicity *in vivo.*

### Umb suppresses RANKL-induced osteoclast differentiation and bone resorption function *in vitro*

**Fig 3:**
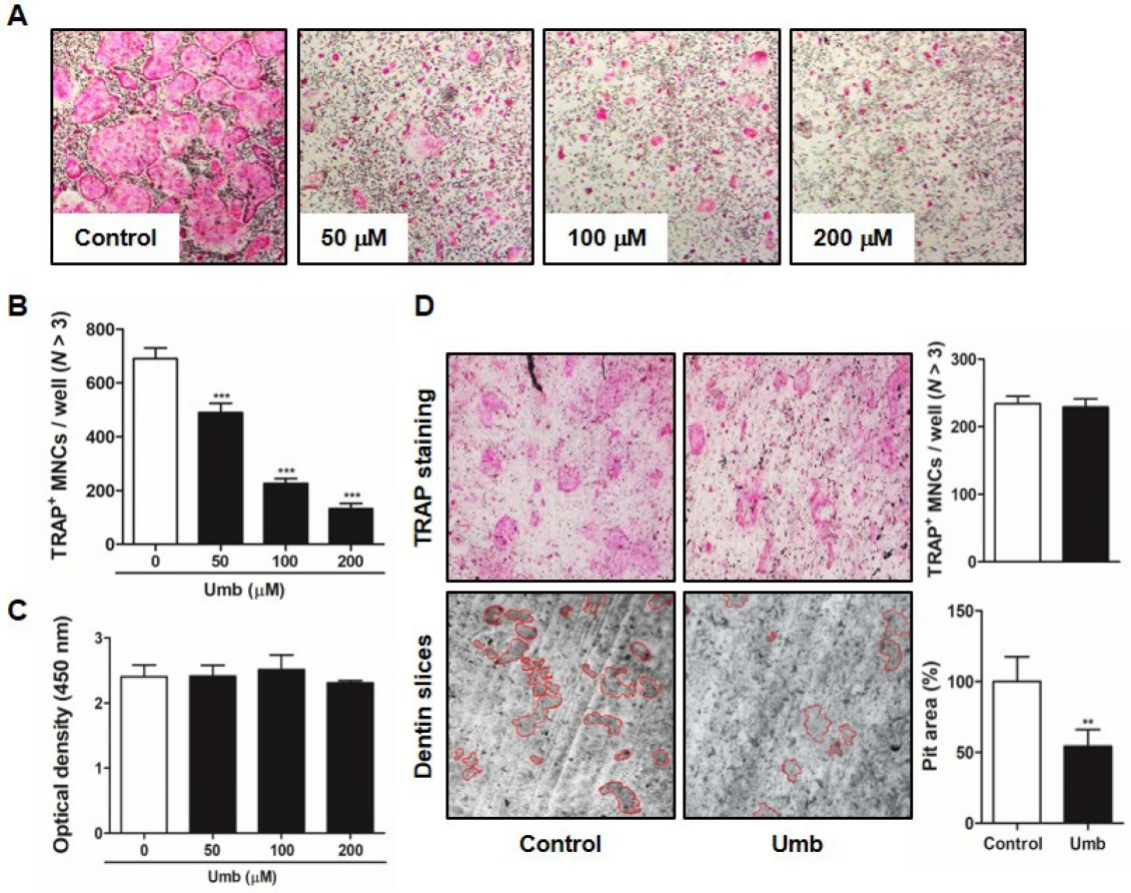
Umb attenuates RANKL-induced osteoclast differentiation and bone resorption *in vitro*. (A) BMMs were cultured for 4 days in the presence of M-CSF (30 ng/mL) and RANKL (100 ng/mL) with the control (DMSO) or Umb. Cells were fixed with 3.7% formalin, permeabilized with 0.1% Triton X-100, and stained with TRAP solution. (B) TRAP-positive MNCs counted as osteoclasts. (C) BMMs were cultured for 3 days at the indicated doses of Umb in the presence of M-CSF (30 ng/mL). Cell viability was determined by XTT assay. (D) Mature osteoclasts were seeded on dentin slices and treated for 48 h with Umb (200 μM). Thereafter, surviving osteoclasts were detected by TRAP staining (*upper left*) and TRAP-positive MNCs were counted (*upper right*). Attached cells were removed from the plates and photographed under a light microscope. Pit areas are lined in red (*lower left*). Pit areas were quantified using ImageJ (*lower right*). ^**^P < 0.01, ^***^P < 0.001 versus the control.

To identify the influence of Umb on RANKL-induced osteoclastogenesis, BMMs were incubated with M-CSF and RANKL in the various concentrations of Umb. Increasing concentrations of Umb exerted dose-dependent inhibition of the formation of TRAP-positive MNCs (Fig. 3A, B). We evaluated the XTT assay to determine whether the inhibitory effect of Umb on osteoclastogenesis was due to reduced viability or proliferation of the osteoclast precursor cells. We determined that Umb did not affect cell viability, even at the highest concentration administered (Fig. 3C). Next, we analyzed the effect of Umb on the function of mature osteoclasts using dentin slices. The area of dentin slice resorption was markedly reduced in the Umb treatment group (200 μM) compared to the control group (Fig. 3D, *lower*). Using TRAP staining, we also confirmed that the anti-resorptive action of Umb did not exert any cytotoxicity on mature osteoclasts (Fig. 3D, *upper*).

### Umb inhibits RANKL-induced Akt signaling pathway

**Fig 4:**
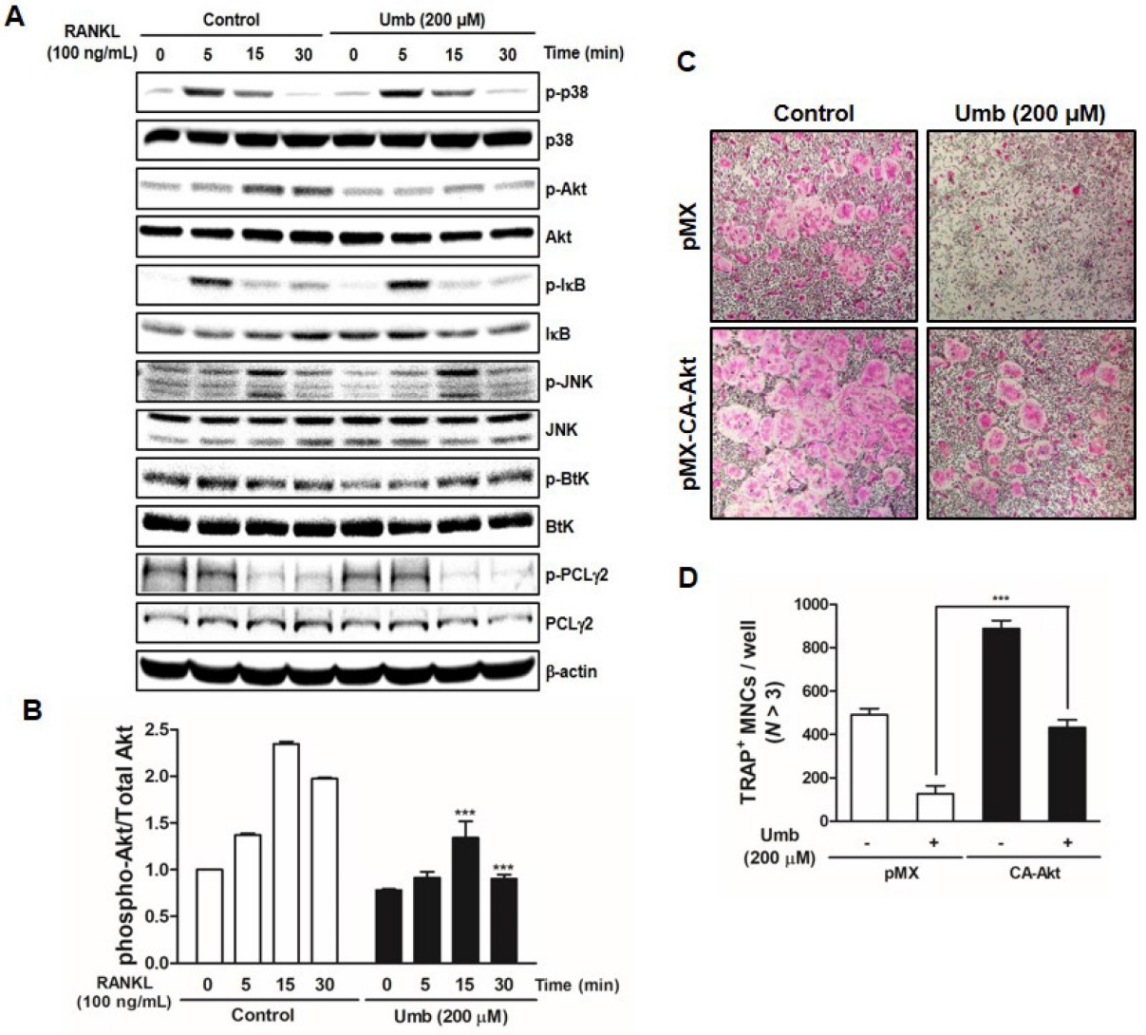
Umb suppresses RANKL-induced Akt signaling pathway. (A) BMMs were pretreated with the control (DMSO) or Umb (200 μM) for 1 h and then stimulated with RANKL (100 ng/mL) for the indicated time. Whole-cell lysates underwent western blot analysis with the indicated MAPK antibodies. β-actin served as the internal control. (B) Representation of the ratio of phosphorylated Akt to total Akt. ^***^P < 0.001 versus the control at indicated times. (C) BMMs were infected with retroviruses expressing pMX-IRES-EGFP (pMX) and pMX-CA-Akt-EGFP. The infected BMMs were cultured with or without Umb (200 μM) in the presence of M-CSF (30 ng/mL) and RANKL (100 ng/mL) for 4 days. Thereafter, the cells were fixed and stained with TRAP solution. (D) TRAP-positive MNCs counted as osteoclasts.

To investigate the mechanism underlying Umb-mediated inhibition of osteoclastogenesis, we tested the effect of Umb on the phosphorylation of early cellular transducers, including mitogen-activated protein (MAP) kinase (p38 and JNK), Akt, IκB, Btk, and PLCγ2. As shown in Figure 4A and B, Umb inhibits RANKL-induced phosphorylation of Akt, but not the phosphorylation of p38, JNK, IκB, Btk, and PLCγ2. To further investigate this mechanism, we used a retroviral transfection-based assay to induce overexpression of Akt in BMMs. The ectopic expression of Akt was sufficient to reverse the suppressive effect of Umb on the formation of TRAP-positive osteoclasts (Fig. 4C and D). These results indicate that Umb inhibits the Akt phosphorylation signaling pathway in the early stages of osteoclast differentiation.

### Umb downregulates RANKL-induced c-Fos and NFATc1 protein expression and stability, as well as osteoclastogenesis-related genes downstream of NFATc1

As key regulators of RANKL-induced osteoclastogenesis, c-Fos and NFATc1 upregulate osteoclast marker genes, such as *OSCAR*, *TRAP*, *ATP6v0d2,* and *CtsK*, during osteoclastogenesis. To assess whether Umb has any effects on the induction of c-Fos and NFATc1, the mRNA and protein expression levels of c-Fos and NFATc1 were examined.

**Fig 5:**
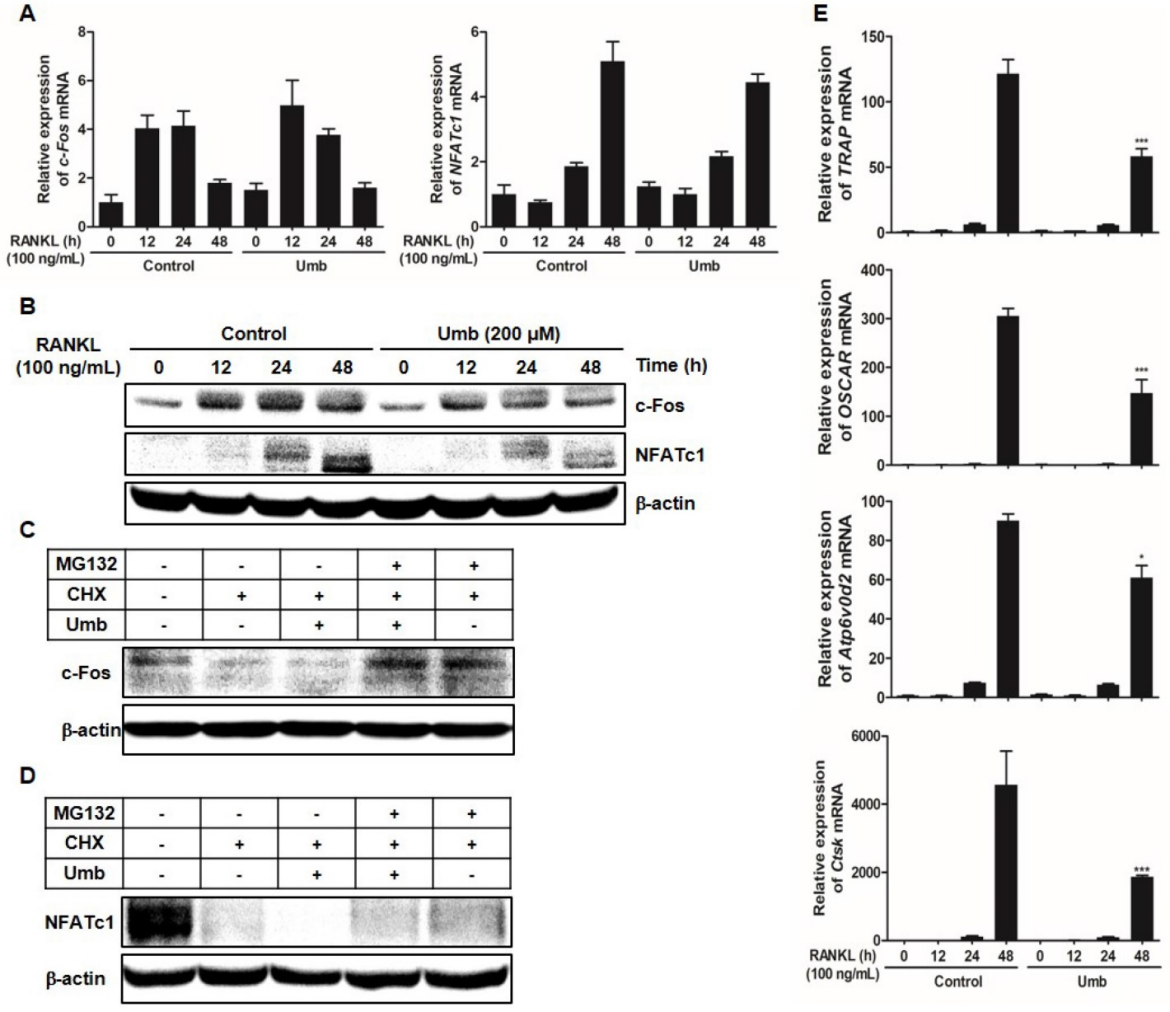
Umb downregulates RANKL-induced c-Fos and NFATc1 protein expression and stability and osteoclastogenesis-related genes downstream of NFATc1. (A) BMMs were stimulated with M-CSF (30 ng/mL) and RANKL (100 ng/mL) in the presence or absence of Umb (200 μM) for the indicated times. The mRNA expression levels of the c-Fos and NFATc1 genes were evaluated by real-time RT-qPCR. (B) Effects of Umb on protein expression levels of c-Fos and NFATc1 were evaluated by western blot analysis. (C) BMMs were infected with pMX-c-Fos-IRES-EGFP (c-Fos) or NFATc1. Infected BMMs were pretreated with or without Umb (200 μM) in the presence of M-CSF (30 ng/mL) for 24 h and then stimulated with RANKL (100 ng/mL). After 20 h, 2 μg/mL CHX and 5 μM MG132 were added to the cultures 4 h before harvest. (D) BMMs were pretreated with or without Umb (200 μM) in the presence of M-CSF (30 ng/mL) for 24 h and then stimulated with RANKL (100 ng/mL). After 48 h, 2 μg/mL CHX and 5 μM MG132 were added to the cultures 4 h before harvest. Western blot analysis was then performed on the cells. Whole-cell lysates underwent western blot analysis with the indicated antibodies. β-actin was used as the internal control. (E) BMMs were pretreated with or without Umb (200 μM) for 1 h and with RANKL (100 ng/mL) for the indicated times. The expression of *TRAP*, *OSCAR*,* ATP6v0d2*, and* CtsK* mRNA was analyzed using real-time RT-qPCR. ^*^P < 0.05, ^***^P < 0.001 versus the control at 48 h.

As shown in Figure 5A and B, both mRNA and protein levels of c-Fos and NFATc1 increased in response to RANKL. Umb significantly inhibited c-Fos and NFATc1 at the protein level, without producing any marked changes in c-Fos and NFATc1 mRNA expression (Fig. 5A, B). To investigate the mechanism of the inhibitory effect of Umb at the protein level, CHX, an inhibitor of protein synthesis, and MG132, an inhibitor of the proteasome, were used to determine whether Umb induces protein degradation of c-Fos and NFATc1. After 48 hours post-transfection with c-Fos or NFATc1, treatment with CHX decreased the expression of c-Fos and NFATc1 proteins compared to the control, and additional Umb treatment accelerated the decrease of c-Fos and NFATc1 protein levels. Moreover, the effect of Umb was reversed by co-treatment with MG132 (Fig. 5C, D). These results suggest that Umb inhibits c-Fos and NFATc1 protein expression and that this mechanism is responsible for protein degradation. Additionally, Umb also significantly downregulated genes downstream of NFATc1, such as *TRAP*,* OSCAR*,* ATP6v0d2* and *CtsK* (Fig. 5E), during osteoclastogenesis.

### Umb did not stimulate osteoblastic differentiation

**Fig 6:**
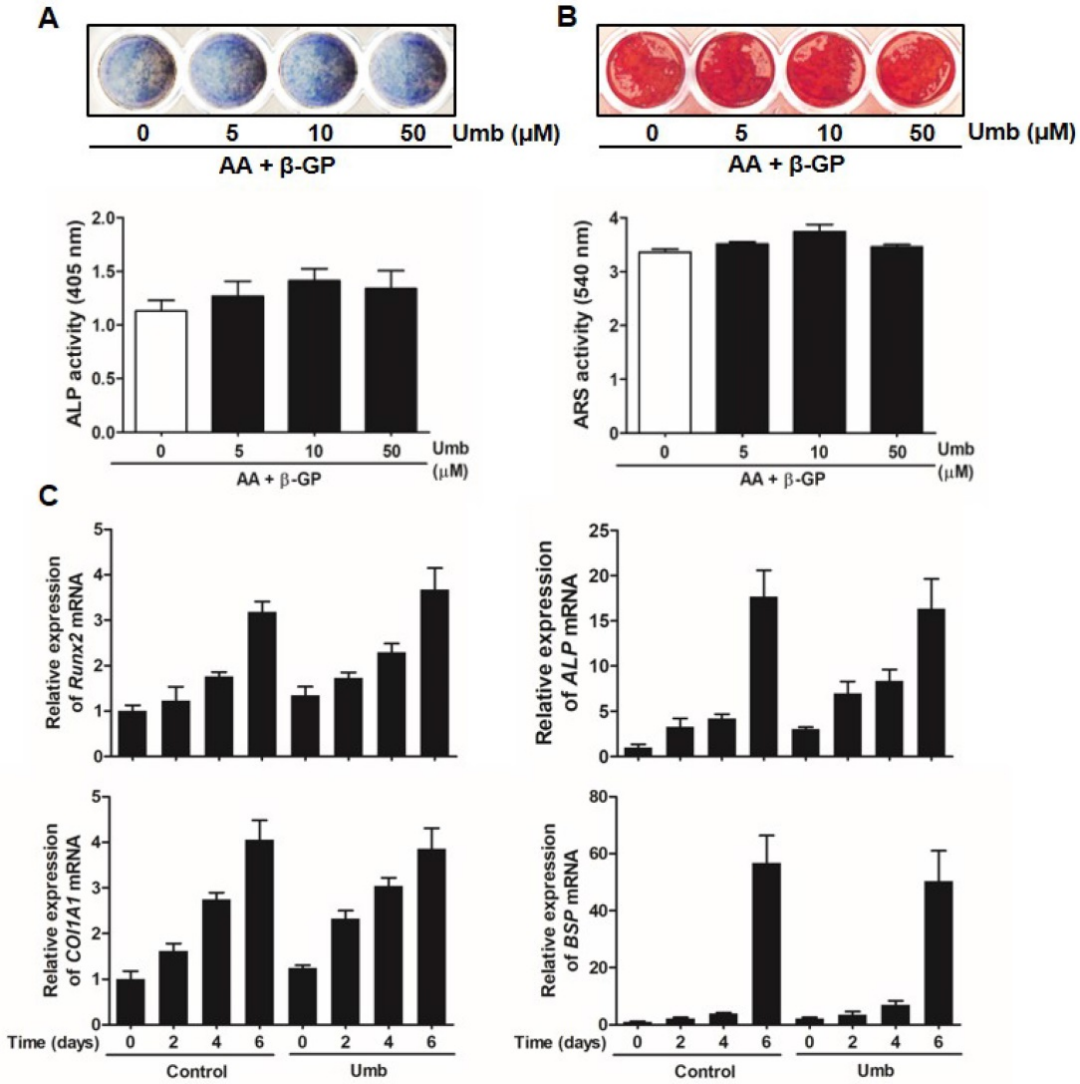
Umb did not stimulate osteoblastic differentiation. (A) Primary osteoblasts were cultured in the presence of AA (50 μg/mL), β-GP (10 mM), and different amounts of Umb for 6 days during osteoblastic differentiation. ALP positive cells were monitored by ALP staining (*upper*). ALP activity is expressed as *p*-nitrophenol released (*lower*). (B) Calcium deposits for matrix mineralization were measured by Alizarin Red S (ARS) staining after 21 days (*upper*). The intensity of staining was quantified with cetylpyridinium chloride (*lower*). (C) The mRNA expression levels of* Runx2*,* ALP*,* COL1A1*, and* BSP* were analyzed by real-time RT-qPCR.

To determine whether Umb influenced osteoblastic differentiation, we examined the ALP and ARS staining and activity assays of primary osteoblasts. As shown in Figure 6A, Umb did not significantly alter the levels of ALP expression and activity. The level and intensity of ARS staining, indicative of mineralization during osteoblastic differentiation, were not stimulated by treatment with Umb (Fig. 6B). Moreover, Umb did not alter the expression of *Runx2*, *ALP*, *COL1A1*, and *BSP*, genes related to osteoblastic differentiation (Fig. 6C). These results suggest that Umb did not affect osteoblastic differentiation.

## Discussion

Several investigations have shown that Umb possesses diverse pharmacological properties including antioxidant 16-18 and anti-inflammatory effects 18-20. However, the role of Umb in biological properties on bone metabolism, particularly osteoclast differentiation and bone resorbing function has not been well studied. To our knowledge, this study is the first demonstration that Umb suppress RANKL-induced osteoclast differentiation, bone resorption, and LPS-induced bone loss *in vivo*.

LPS is known to be a common trigger of bone loss accompanying enhanced osteoclastic activity 6-9. The pathological bone conditions initiated by LPS exhibit the recruitment of a number of cell types, such as macrophages, fibroblasts, and osteoclasts, followed by the induction of various pro-inflammatory cytokine secretions, including IL-1, IL-6, TNF-α, and M-CSF 6-9. These pro-inflammatory cytokines subsequently trigger the formation of bone resorbing mature osteoclasts from osteoclast precursors. Furthermore, RANKL contributes to osteolysis by promoting exacerbated osteoclast activity and accelerating bone resorption in response to inflammatory cytokines 21. Given the crucial role of excessive osteoclastic activity in bone destruction, the development of osteoclastic inhibitors can offer valuable therapies for the prevention and treatment of pathological inflammatory bone loss. We identified clear beneficial effects of Umb on the structure, density, and osteoclast number of femur trabecular bones in mice* via* micro-CT (Fig. 1) and histological analyses (Fig. 2), confirming the *in vivo* efficacy of Umb in diseases involving inflammatory bone loss.

When RANKL interacts with RANK, it initiates a cascade of intracellular signaling, including MAPK, Akt, NF-κB, Ca^2+^, c-Fos, and NFATc1, which are crucial for osteoclastogenesis 10, 11, 22, 23. Among these signaling pathways, the RANKL/RANK axis results in the robust induction of transcription factors c-Fos and NFATc1, which play an indispensable role in osteoclast differentiation. As a component of activator protein-1 (AP-1) transcription factor complex, c-Fos is involved in the regulation of osteoclast formation 22, 23. Targeted disruption of c-Fos in mice induces osteopetrosis, characterized by a lack of osteoclasts 24. In addition, ectopic expression of NFATc1 eliminates the requirement of RANKL for mature osteoclast differentiation, even in the absence of RANKL 12. NFATc1 knockout embryonic stem cells do not undergo osteoclastogenesis successfully in response to RANKL, suggesting that NFATc1 plays an essential role in RANKL-induced osteoclastogenesis 12. In this study, Umb was shown to markedly downregulate the protein levels of c-Fos and NFATc1 without any marked changes in c-Fos and NFATc1 mRNA expression (Fig. 5A, B). Our protein stability assay showed that downregulation of c-Fos and NFATc1 by Umb was reversed by treatment with MG132, a proteasome inhibitor (Fig. 5C, D). These results suggest that Umb decreases the stability of c-Fos and NFATc1 proteins *via* proteasome degradation, thereby inhibiting the transcriptional activity of c-Fos and NFATc1. Once NFATc1 activation is induced downstream, osteoclast-specific genes are upregulated, such as *TRAP, OSCAR, ATP6v0d2,* and *CtsK* 25, 26. Our studies found that Umb significantly attenuated mRNA levels of osteoclast marker genes (Fig. 5E). The RANKL/RANK axis also activates early signaling cascades, including the phosphorylation of MAPK, Akt, NF-κB, Btk, and PLCγ2 10, 11, 22, 23. Among the RANKL-dependent signal transducers, Akt in BMMs is known to play a critical role in osteoclast differentiation and survival. It was reported that Akt deficiency abrogates osteoclastogenesis by controlling the nuclear localization of NFATc1 in response to decreased expression of RANKL *in vitro* 27, 28. A pathological phenotype including impaired bone development and dwarfism is observed in Akt knockout mice 29. Additionally, the clinical importance of Akt was demonstrated by elucidating the therapeutic action of the systemic administration of the Akt inhibitor LY294002 on multiple myeloma-mediated osteoclast formation in severe combined immunodeficiency mice 30. From our results, we determined that Umb inhibits the RANKL-induced phosphorylation of Akt, but not the phosphorylation of p38, JNK, IκB, Btk, and PLCγ2 (Fig. 4A and B). Furthermore, the forced expression of CA-Akt rescued the Umb-induced inhibition of osteoclast differentiation, suggesting that the downregulation of Akt is responsible for the inhibitory effect of Umb (Fig. 4C and D).

Taken together, our studies indicate that Umb inhibits the RANKL-induced Akt-c-Fos-NFATc1 signaling pathways, which leads to the inhibition of osteoclast differentiation and function *in vitro*. Moreover, Umb protects against LPS-induced osteolysis *in vivo*, congruent with its effects *in vitro*. Therefore, Umb exhibits potential therapeutic effects against osteoclast-related osteolytic disease.
